# Knowledge, attitudes and preferences of parents/guardians regarding dental treatment of their children’s primary teeth: a questionnaire cross-sectional study

**DOI:** 10.1007/s40368-025-01002-z

**Published:** 2025-01-31

**Authors:** C. Ninou, K. Seremidi, A. Agouropoulos, W. Papaioannou, S. Gizani

**Affiliations:** 1 Department of Paediatric Dentistry, School of Dentistry, National and Kapodistrian University of Athens, Athens, Greece; 2 Department of Preventive and Community Dentistry, School of Dentistry, National and Kapodistrian University of Athens, Athens, Greece

**Keywords:** Oral health, Parental knowledge, Parental attitudes, Parental acceptance, Dental treatment, Silver diamine fluoride

## Abstract

**Purpose:**

Assess the knowledge and attitudes of parents/guardians regarding their children's oral health and their preferences regarding the treatment of carious primary teeth.

**Methods:**

A cross-sectional study including the completion of a questionnaire by parents/guardians of healthy children aged 2–12 years attending the Department of Pediatric Dentistry (NKUA). Data were collected through 35 questions regarding demographic characteristics of the participants, oral hygiene and dietary habits, and parents’ knowledge of the importance of oral health and primary teeth. Treatment preferences were assessed through clinical scenarios accompanied by colored clinical photographs. Results were presented in frequency tables and comparisons with specific child and parent-characteristics were evaluated using chi-squared and Fisher's exact test.

**Results:**

Parents were mainly females (73%), married (81%), aged > 40 years (56%), and high school graduates (57%). Most children brushed alone (56%), twice (48%) daily, with a manual toothbrush (65%) and consumed sugary snacks daily (38%). More than two-thirds of parents recognized the relationship between oral hygiene and general health (82%) and the importance of primary teeth (72%). Almost all parents would like to restore their children’s asymptomatic (94%) and symptomatic (98%) primary teeth, with pulp therapy and stainless-steel crowns being the treatment of choice (58%). The decision on treatment about cavitated/non-cavitated primary teeth was not correlated with any of the parental or child-related factors. Acceptance of minimally invasive treatments was higher among highly educated parents and parents of boys.

**Conclusion:**

Insight into parents' perceptions and preferences regarding the treatment of primary teeth is necessary and should be considered during treatment planning.

## Introduction

In recent years, medical/dental care has shifted to a patient-centered approach which is defined as ‘care provision that is consistent with the values, needs, and desires of patients and is achieved when clinicians involve patients in healthcare discussions and decisions’ (Constand et al. 2014). Key components of patient-centered care are communication, partnership and health promotion. One should not ignore that parents are responsible for consenting to any dental treatment after they have been thoroughly informed of the available treatment options. Their decision is greatly affected by their knowledge and perceptions regarding the oral health status of their children (Schroth et al. 2007) and can greatly enhance patient-reported outcomes and satisfaction.

Parental knowledge of oral health differs widely around the world, with studies reporting on parental attitudes regarding primary teeth showing controversial results. There are studies reporting that many parents believe that primary teeth are not important and do not require dental treatment (Sheetal et al. 2022; Al-Batayneh et al. 2019) while in others, parents clearly support their importance (Hussein et al. 2013) and need for treatment (Vittoba Setty and Srinivasan 2016). These differences have been attributed to the variations in the beliefs and cultures of the different populations, which advocate that primary teeth are replaceable and that their loss, even prematurely, is considered normal and harmless (Casamassimo 2003).

Beliefs regarding decision-making on dental treatment for children have also been investigated with most studies showing that parents report that clinicians should have the final decision, especially when it comes to more difficult, complicated and expensive treatments (Murdoch et al. 2023; Benecke et al. 2020; Adewumi et al. 2001). Decision-making ability is also affected by factors such as past experiences and general knowledge, as they may support sufficient understanding. Competence to consent is directly related to one’s ability to understand the purpose, the risk and the benefits of the proposed treatment and involves comprehension of provided information and weighing up the information (Adewumi et al. 2001).

To the best of our knowledge, there is limited evidence of parental acceptance of dental treatment for their children, with most focusing on behavioral management techniques and treatment under general anesthesia or conscious sedation with nitrous oxide (Al-Batayneh et al. 2019). The ones assessing parental attitude regarding treatment reported a percentage that exceeds 40% for accepting clinicians’ decision even in cases where pulp therapy and restorative treatment were proposed (Hussein et al. 2013; Popoola et al. 2013; Tickle et al. 2003). Acceptance levels and compliance with clinician’s opinions were high even when the treatment proposed was the extraction of a symptomatic primary tooth in children aged 2–12 years in Jordan and Malaysia (Sheetal et al. 2022; Al-Batayneh et al. 2019; Tickle et al. 2003).

The use of Silver Diamond Fluoride (SDF) is probably the most widely used and well-documented minimally invasive technique. Evidence regarding its acceptance is controversial, with some studies showing that the technique is acceptable by the majority of parents, especially for children under the age of 6 or at high caries risk (Kumar et al. 2019), with most parents positively appraising the ease of application and the painlessness of the procedure (Clemens et al. 2018). Other studies though reported that there is a remarkable percentage of parents that strongly refused the application of SDF because of the black staining (Alshammari et al. 2019; Bagher et al. 2019), with the authors stating that parents of uncooperative children were more positive about this type of treatment. Although, the correlation between parental attitude and treatment preferences and factors such as their age, knowledge about oral hygiene, caries and caries prevention has not yet been fully investigated (Tickle et al. 2003).

The heterogeneity in the parental knowledge questionnaires and the limited studies on specific parental preferences regarding the treatment of their children's primary teeth, especially on "traditional" treatment options, demonstrate the need for further studying. Detailed assessment of parental knowledge, beliefs, and treatment preferences is necessary to provide a comprehensive input of parental expectations regarding dental treatment and to facilitate shared decision making.

The aim of the present study was to assess the knowledge and the attitudes of parents/guardians in Greece regarding their children’s oral health and their preferences regarding the type of treatment for carious primary teeth. A secondary objective was to correlate knowledge and acceptance with specific parental and patient characteristics. Therefore, the Null hypothesis states that parents/guardian’s knowledge regarding children’s oral health is poor and their preferences regarding treatment is not affected by patient and parent-related characteristics.

## Materials and methods

### Study design

The present was a cross-sectional study that involved the completion of an anonymous structured questionnaire by parents/guardians regarding knowledge and acceptance of the treatment of children’s primary teeth. The research protocol was submitted and approved by the Ethics Committee of 'School of Dentistry, National and Kapodistrian University of Athens (NKUA)’ (N556/Approved on 01.03.2023).

### Sample

The sample was derived from the patients attending the Under and Post-graduate clinics of the 'Paediatric dentistry department, NKUA’. The inclusion criteria were parents/guardians of healthy children, aged 2–12 years, who require or have received restorative dental treatment on primary teeth, and who could read and understand the Greek language. Exclusion criteria included parents/guardian of children younger than 2 years or older than 12 years of age, with a contributory medical history, who have not received any dental treatment or only needing preventive care and parents/guardian who do not understand Greek. Eligible participants to be included were randomly selected using random numbers allocated by a third person not involved in the study.

The sample size was calculated using the equation *n* = n0/Ν, where N represents the total number of patients attending the above clinics for treatment during a four-month period and was equal to 2000. Respectively, n0 = Z 2 1-a/2 p(1-p)/e 2 and was calculated equal to 271, since Z 2 1-a/2 was set at 1.65, significance level a = 10%, *p* = 0.5 and the highest accepted error e = 0.05. Considering a 10% non-respondent rate, the final sample was calculated to be 280 participants.

### Data collection

Data were collected through a self-administered questionnaire consisting of 35 multiple-choice questions divided into five sections. It was constructed based on similar questionnaires previously used in other studies and aimed to qualitatively assess the knowledge of parents on their children’s oral health and the importance of primary teeth and their attitudes towards the treatment of cavitated and non-cavitated teeth.

**Fig. 1:**
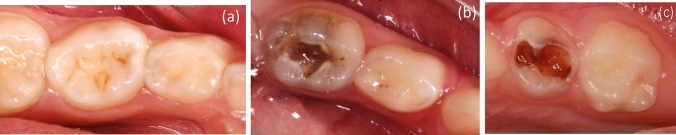
Clinical pictures accompanying scenarios regarding treatment of a: **a** non-cavitated, asymptomatic primary molar, **b** cavitated, symptomatic primary molar and **c** cavitated, asymptomatic primary molar in a child with difficulties in co-operation

Data collected involved: a. demographic characteristics of both children and parents/guardians, b. oral hygiene and dietary habits, c. parental knowledge, through questions on the effect of oral health on general health and the importance of primary dentition and d. parental priorities and preferences for the treatment of primary teeth, using three cases with clinical photographs. In the first case, participants could choose to treat or monitor a non-cavitated, asymptomatic carious primary molar, in the second they were asked to choose between two proposed treatment options (pulp therapy and a SSC or extraction and placement of a space maintainer) for the treatment of a symptomatic cavitated carious primary molar and in the third acceptance of SDF application was assessed for the treatment of an asymptomatic, cavitated primary molar, in a non-cooperative patient (Fig. 1).

The questionnaire was distributed in paper form and collected in the waiting room during the children's scheduled appointments in the clinics between January and April 2023. Completion of the questionnaire was anonymous and only one parent per family could participate in the study. If a family had more than one child who met the inclusion criteria, the parent randomly selected the child for whom he/she would complete the questionnaire.

Prior to study initiation, the questionnaire was tested for clarity and accuracy on a random sample of 20 parents/guardians, that were not included in the final sample.

### Statistical analysis

Data were collected into an Excel spreadsheet and analyzed using SPSS Version 21.0 (SPSS Inc, Chicago, IL, USA). Descriptive statistics were used to determine oral health-related behaviors and knowledge and the differences in the distribution of the answers for specific sample characteristics (parental and children’s age, parental educational level and family status) were tested using chi-square and Fisher’s exact tests. Differences in parental acceptance and preferences regarding dental treatment of primary teeth were tested using chi-square test and multivariate regression analysis was used to present the probability of accepting options of SDF application as a minimally invasive treatment option according to variables associated with patient and parent characteristics. Statistical significance was set at *p* < 0.05.

## Results

### Sample

From the total pool of patients attending the Paediatric Dentistry department during a period of four months, the majority required or had undergone dental treatment (70%). The participants initially excluded were parents/guardian of children outside the age range set (5%), children with a medical condition that directly affects oral health (10%), that visited the clinic for the first time for prevention (15%) and parents/guardian with language barriers (5%). Of the 264 parents/guardians who completed the questionnaire (response rate: 94%), 73% were females of which 56% were older than 40 years and 74% were married. More than half (57%) had completed secondary education and 40% lived in the center of Athens. Respectively, 133 children were boys and 131 girls, with a mean chronological age of 8.09 years (s.d. 0.14).

### Oral health-related behavior

Almost half of the parents (48%) reported that their children brushed their teeth twice daily, with 27% reporting brushing only at night. Fifty-six percent reported brushing alone, and 21% under supervision. The majority of children (65%) used manual toothbrush and 52% of parents didn’t know the fluoride concentration of the toothpaste used. There were no statistically significant differences for the above variables between parents with different age, educational level and family status (Table 1). Regarding dietary habits, the majority of children consumed sugary snacks daily (38%), with parents of lower educational level reporting significantly higher consumption (*p* = 0.01).

**Table 1:** Distribution of oral hygiene habits according to parental characteristics

	Age N(%)			Educational level N(%)				Family status N(%)		
	< 40 yrs	> 40 yrs	p-value	Primary	Secondary	Higher	p-value	Both parents	Single parent	p-value
Brushing frequency										
Twice daily	53 (42)	73 (58)	*0.213*	9 (7)	66 (52)	51 (41)	*0.434*	98 (78)	28 (22)	*0.334*
Once daily	54 (51)	52 (49)		6 (6)	60 (56)	40 (38)		73 (69)	33 (31)	
Sporadically	12 (37)	20 (63)		2 (6)	23 (72)	7 (22)		24 (75)	8 (25)	
Parental role in tooth brushing										
None	68 (46)	79 (54)	*0.342*	10 (7)	82 (56)	55 (37)	*0.442*	105 (71)	42 (29)	*0.521*
Supervision	20 (36)	35 (64)		1 (2)	30 (54)	24 (44)		44 (80)	11 (20)	
Brushing	31 (50)	31 (50)		6 (10)	37 (60)	19 (30)		46 (74)	16 (26)	
Toothbrush										
Manual	83 (48)	90 (52)	*0.235*	13 (8)	96 (55)	64 (37)	*0.321*	129 (75)	44 (25)	*0.221*
Powered	20 (45)	24 (55)		3 (7)	21 (48)	20 (45)		28 (64)	16 (36)	
Both	16 (34)	31 (66)		1 (2)	32 (68)	14 (30)		38 (81)	9 (19)	
Toothpaste										
Non-fluoridated	14 (64)	8 (36)	*0.112*	3 (14)	11 (50)	8 (36)	*0.315*	12 (55)	10 (45)	*0.114*
Fluoridated	40 (38)	64 (62)		5 (5)	54 (52)	45 (43)		79 (76)	25 (24)	
Don’t know	65 (47)	73 (53)		9 (6)	84 (61)	45 (33)		104 (75)	34 (25)	

### Oral health-related knowledge

Eighty-two percent of the parents believed that oral health affects general health and 72% rated primary teeth as being very important. The higher the parental education, the more important they considered primary teeth to be (*p* = 0.033). Regarding possible aetiological factors for dental caries, 6% reported infrequent tooth brushing, 21% frequent consumption of sweets and 51% combination of both. No statistically significant association was found between the above and any of the parent-related characteristics (*p* > 0.05).

### Preferences regarding dental treatment

Regarding the acceptance of proposed treatment plans, 79% percent responded that they would immediately accept the treatment recommended by their dentist, 6% would seek a second opinion and 15% would discuss other alternatives. This high acceptance rate was not significantly affected by any of the parental-related characteristics.

Fathers were more concerned than mothers about the length of the appointment (*p* = 0.045), the time until exfoliation of the primary tooth (*p* = 0.024) and the cost of the treatment option (*p* = 0.044). In addition, aesthetics was more important to older parents (*p* = 0.05). Parents with higher education were significantly less concerned about the duration of the appointment (*p* = 0.018) and more concerned about pain sensation (*p* = 0.021) (Table 2).

**Table 2:** Parental priorities in decision-making according to their specific characteristics (* indicating statistical significance)

	Duration of appointment N(%)	Total number of appointments N(%)	Cost N(%)	Remaining time until exfoliation N(%)	Co-operation N(%)	Potential pain sensation N(%)	Aesthetics N(%)
Parental gender							
Male	23 (32)*	10 (14)	17 (24)*	6 (9)*	35 (49)	21 (30)	7 (10)
Female	41 (21)	24 (12)	27 (14)	37 (19)	104 (54)	63 (33)	18 (9)
Parental age							
< 40yrs	32 (27)	14 912)	15 (13)	20 (17)	65 (55)	41 (35)	7 (6)
> 40 yrs	32 (22)	20 (14)	29 (20)	23 (16)	74 (51)	43 (30)	18 (12) *
Parental educational level							
Primary	4 (24)	4 (24)	0 (0)	3 (18)	8 (47)	2 (12)	0 (0)
Secondary	46 (31)	19 (13)	24 (16)	29 (20)	79 (53)	42 (28)	15 (10)
Higher	14 (14) *	11 (11)	20 (20)	11 (11)	52 (53)	40 (41) *	10 (10)
Family status							
Both parents	54 (28)	26 (13)	39 (20)	28 (14)	107 (55)	60 (31)	16 (8)
Single parent	10 (14)	8 (12)	5 (7)*	15 (22)	32 (46)	24 (35)	9 (13)

Tables 3 and 4 present the answers on the choice of treatment for the three clinical cases. For the case of the asymptomatic tooth, 94% would choose to restore the tooth, but older parents were more likely to refuse treatment because it was a primary tooth (*p* = 0.05). For the case of the symptomatic tooth most parents would choose to restore the tooth (98%), with vital pulp treatment and restoration with an SSC being the treatment of choice in 58% of the answers. The above differences were not statistically significant (Table 3).

**Table 3:** Treatment acceptance for the non-cavitated, asymptomatic primary molar (Case 1) and the cavitated, symptomatic primary molar (Case 2), according to parents’ and children’s characteristics

	Case 1					Case 2				
	Treatment N(%)		Reason for not N(%)			Treatment N(%)		Criteria of choice N(%)		
	Yes	No	Deciduous tooth	Symptomless	Treat another tooth	Pulpotomy + Stainless steel crown	Extraction + Space maintenance	Treatment-related	Co-operation	Aesthetics
Parental gender										
Male	66 (93)	5 (7)	1 (20)	1 (20)	3 (60)	38 (58)	28 (42)	14 (21)	39 (60)	12 (19)
Female	182 (94)	11 (6)	3 (27)	5 (46)	3 (27)	107 (59)	75 (41)	29 (18)	104 (64)	30 (18)
Parental age										
< 40 yrs	112 (94)	7 (6)	0 (0)*	3 (43)	4 (57)	67 (61)	42 (39)	22 (21)	64 (62)	17 (17)
> 40yrs	136 (94)	9 (6)	4 (44)*	3 (34)	2 (22)	78 (56)	61 (44)	21 (17)	79 (63)	25 (20)
Parental educational level										
Primary	14 (82)	3 (18)	0 (0)	2 (67)	1 (33)	8 (53)	7 (47)	3 (21.5)	8 (57)	3 (21.5)
Secondary	142 (95)	7 (5)	1 (14)	3 (43)	3 (43)	81 (57)	61 (43)	26 (20)	80 (63)	22 (17)
Higher	92 (94)	6 (6)	3 (50)	1 (17)	2 (33)	56 (61)	35 (39)	14 (16)	55 (64)	17 (20)
Family status										
Both	180 (92)	15 (8)	4 (27)	6 (40)	5 (33)	115 (61)	74 (39)	37 (20)	114 (63)	31 (17)
Single	68 (98.5)	1 (1.5)	0 (0)	0 (0)	1 (100)	30 (51)	29 (49)	6 (13)	29 (63)	11 (24)
Gender of child										
Boy	124 (93)	9 (7)	2 (22)	2 (56)	2 (22)	76 (60)	50 (40)	18 (16)	71 (62)	25 (22)
Girl	124 (95)	7 (5)	2 (29)	1 (14)	4 (57)	69 (57)	53 (43)	25 (22)	72 (63)	17 (15)
Age of child										
Primary	59 (92)	5 (8)	2 (40)	1 (20)	2 (40)	40 (68)	19 (32)	8 (17)	4 (8)	35 (75)
Early mixed	86 (97)	3 (3)	1 (33)	2 (67)	0 (0)	46 (55)	37 (45)	7 (11)	3 (5)	55 (84)
Late mixed	103 (93)	8 (7)	1 (12)	3 (38)	4 (50)	59 (56)	47 (44)	13 (17)	8 (11)	53 (72)
Importance of primary teeth										
None	7 (88)	1 (12)	0 (0)	1 (100)	0 (0)	4 (50)	4 (50)	2 (25)	5 (63)	1 (12)
Low	58 (89)	7 (11)	3 (43)	4 (43)	1 (14)	36 (59)	25 (41)	13 (23)	31 (54)	13 (23)
High	183 (96)	8 (4)	1 (12)	2 (25)	5 (63)	105 (59)	74 (41)	28 (18)	107 (66)	28 (17)
Previous dental treatment										
Prevention	38 (95)	2 (5)	1(50)	0 (0)	1 (50)	23 (59)	16 (41)	9 (25)	21 (58)	6 (17)
Restorative	190 (95)	10 (5)	1(10)	6 (60)**	3 (30)	109 (58)	78 (42)	28 (16)	114 (66)	30 (18)
No treatment	20 (83)	4(17)	2(50)	0 (0)	2 (50)	13 (59)	9 (41)	6 (30)	8 (40)	6 (30)

^*^statistical significance p < 0.05, **statistical significance p < 0.1, as calculated using chi-square and Fisher’s exact tests

**Table 4:** Multivariate analysis for the acceptance of SDF application, using Pearson correlation coefficient

	Exp (B) Odds Ratio	95% Confidence Interval	Significance
Parental gender			
Male	0.88	0.48–1.63	*0.683*
Female	Ref		
Parental Age			
< 40 yrs	1.28	0.73–2.24	*0.393*
> 40 yrs	Ref		
Parental educational Level			
Primary	0.33	0.11–0.97	*0.051*
Secondary	0.57	0.33–0.99	*0.042*
Tertiary	Ref		
Family status			
Both parents	0.73	0.41–1.32	*0.304*
Single parents	Ref		
Gender of Child			
Boy	1.85	1.12–3.07	*0.022*
Girl	Ref		
Dentition child			
Primary dentition	0.73	0.38–1.4	*0.343*
Early mixed	1.5	0.83–2.7	*0.181*
Late mixed	Ref		

Treatment of the asymptomatic primary tooth, in an uncooperative child, with the application of SDF, was directly accepted by 56% of parents, with 43% reporting that they would prefer treatment with a resin composite restoration. Parents of boys (*p* = 0.013) and of higher educational level (*p* = 0.057) were correlated with greater acceptance of SDF as a treatment option for the carious primary tooth (Table 4).

## Discussion

In the present study, parental knowledge regarding children's oral health-related habits, and their preferences regarding treatment of primary teeth were assessed in an attempt to provide insights into the factors affecting parental decision-making. Results showed increased knowledge of oral health and a high acceptance of the importance of primary teeth, although oral hygiene and dietary habits were not ideal. Regarding dental treatment, in most cases parents would accept the clinician’s recommendations, while the duration of the appointment, the tooth exfoliation time, cost of the treatment, and aesthetics were the factors directly affecting their final decision.

From the study sample, almost half of the children brushed their teeth twice daily with a manual toothbrush and fluoridated toothpaste. In a previous Greek study of preschool children, it was reported that 51% of the children brushed only once daily and 48% brushed alone (Agouropoulos et al. 2013). Supervision of tooth brushing was reported in only 21% of the present study sample, with 20% of children younger than 6.5 years brushing their teeth alone. This might mean that parents in Greece may not consider or insist on supervising their children during tooth brushing. Data from other countries present great variation with low (Nagaveni et al. 2011) or high percentages (Al-Batayneh et al. 2019) for twice daily brushing, while supervised toothbrushing has been reported as relatively high (Gibi et al. 2020; Al-Batayneh et al. 2019).

These differences can be attributed to the variability of the samples and the specific characteristics of the participants. More specifically, Nagaveni et al. (2011) did not report parental characteristics or the age of the children for whom the answers were given, which, as mentioned above, may directly influence the children's habits and therefore reported answers. Al-Batayneh et al. (2019) reported that three-quarters of children brush twice daily, with the high percentage being probably attributed to the higher education level overall of the majority of participants. Parental education level can affect their perception of oral hygiene and consequently children's performance of oral hygiene.

Regarding dietary habits, results from the present study reported that 38% of children consumed sugary snacks on a daily basis, which is lower than the corresponding percentages reported previously for Greece. Kavvadia et al. (2012), found that 83% of children ate more than three sugary snacks per day. The difference can be probably attributed to the difference in age groups of the two studies. In children, for whom decisions are not independent, principal risk factors for caries development, such as diet and oral hygiene, are determined by factors related to family values and lifestyles. These factors in turn are related to parental culture and socio-economic background. Parental education level has been found to be negatively correlated with the consumption of sugary foods (Flores-Barrantes et al. 2022). This was confirmed in our study, as children of parents with primary education had a higher intake of sugary foods compared to their counterparts of higher education.

Most of the respondents considered primary teeth to be very important (72%), with corresponding results from the literature being controversial. Some studies reported similar (Vittoba Setty and Srinivasan 2016; Hussein et al. 2013) and others different results (Al-Batayneh et al. 2019; Nagaveni et al. 2011). Cultural differences, personal beliefs and socioeconomic background are factors that can directly affect this perception and justify the reported variance (Casamassimo 2003). The high rate of acknowledging the importance of primary teeth can be underlined by the increased percentage of responders identifying the importance of the connection between oral and general health, which has been previously documented in Greek parents (Agouropoulos 2012).

The majority of the participants reported that would accept the dentist’s recommendation for the proposed treatment plan, which is in accordance with the literature where it is widely reported that patients prefer the final decision to be made by the dentist (Murdoch et al. 2023; Benecke et al. 2020; Al-Batayneh et al. 2019; Popoola et al. 2013; Tickle et al. 2003; Adewumi et al. 2001). Lack of clinical knowledge makes patients and parents feel uncertain about their decisions, and they prefer to share the responsibility with their doctor (Alsulamy et al. 2021; Schooten et al. 2003), although patient participation in the decision-making process is of great importance for the outcome of the treatment plan. Knowledge of parental concerns and priorities regarding dental treatment and their perception of various aspects of health, helps clinicians to focus on patients’ needs and provide them with adequate information. This is in accordance with the widely accepted approach of shared decision-making in the medical field (Alsulamy et al. 2021).

According to the results of this study, parents prioritize their child's cooperation and potential sense of pain over cost when choosing a treatment option. The School of Dentistry (NKUA) provides dental care to all children at a low cost, making it accessible to families of all socioeconomic backgrounds. This may explain why cost wasn't considered as such an important factor. The results might have been different if the sample had originated from private dental practices. This can be also highlighted in countries where public or private insurances cover part or the total cost of treatment, and therefore patients might choose treatments beyond defined needs (Felgner and Henschke 2023). Previous studies have reported cost, long duration and fear as the main factors parents consider before deciding upon treatment, with some reporting on the effect in regard to prevention and caries treatment (Felgner et al. 2022; Schwendicke and Göstemeyer 2016).

The vast majority of the parents reported that they would have an asymptomatic tooth restored, even if there was no obvious cavity or pain, which is consistent with the high recognition of the importance of primary teeth recorded in the present study. This is in accordance with the study by Al-Batayneh et al. (2019) but different when compared with the results of Sheetal et al. (2022), where a high percentage of parents preferred to deny treatment or wait until pain symptoms appeared. Tickle et al. (2003) reported that nearly one-third of parents chose to monitor and not treat an asymptomatic carious tooth. This non-interventional approach for the treatment of carious primary teeth either indicates a tendency for a reluctance to intervene for "minor symptomless elements" or that dentists may be adopting a more conservative approach to care, with parents accepting this philosophy. The authors clearly stated that neither socio-economic status, reported attendance patterns nor the children’s anxiety levels seemed to significantly affect the decision on treatment. It is worth mentioning that in the same study parents whose children had previously undergone dental treatment, were more likely to express a preference for restorative care.

In the case of the symptomatic primary tooth, although most of the parents would seek treatment, there were four who would not. This can only be attributed to the low socioeconomic status related to these cases. An even higher percentage of refusal (16,8%) has been reported previously in Northern Jordan, where more than half of parents stated that as primary teeth will be replaced there is no need to treat them (Al-Batayneh et al. 2019). Pain is one of the factors that influence oral health-related quality of life in children (Clementino et al. 2015) and it is surprising that the parents would leave painful teeth untreated.

Regarding treatment options, although there were no statistically significant differences according to parental and children’s characteristics, there was a slight preference for restoration compared to extraction. Results in the literature are controversial, with studies indicating a clear preference for extraction over more conservative and less painful procedures due to the immediate pain relief (Gibi et al. 2020; Al-Batayneh et al. 2019; Bozorgmehr et al. 2013; Thakare et al. 2012). Extractions were more likely to be chosen by parents whose children were irregular attenders and who present to the dentist only when in pain, attitudes correlated to low socioeconomic backgrounds (Tickle et al. 2003).

Finally, acceptance of SDF applications reached 56% while in most studies this exceeds 60% (Wajahat et al. 2022; Kumar et al. 2019; Crystal et al. 2017). This could be attributed to the fact that, in Greece, parents and clinicians have limited knowledge and experience in the use of the material. Although very few parents prioritized esthetics when it comes to their children's treatment, it is interesting that 43% preferred a "white filling" probably because of the color after SDF application. Parents of boys were significantly more positive for SDF, which has also been reported previously (Asif and Gurunathan 2020) and may reflect the greater concern of parents about appearance in girls than in boys. It should be noted though, that the results might have been different if the option of “smart technique” had been introduced.

Current dental public systems inevitably face the great problem of contemporary lifestyles that affect oral health and alleviation of dental problem through the unnecessary removal of tooth structure is inevitable. The restorative approach for dental caries management is failing to improve oral health outcomes for many patients. Lately, clinicians endeavor to implement minimally invasive techniques which continue to grow despite some significant challenges that they present they continue to grow. This is well supported by the overall acceptance of parents/guardians, although there is an urgent need to undertake research to assess the cost-effectiveness of this approach and their continual application by clinicians.

The high acceptance of these techniques can be utilized for the planning of new policies towards their adoption them for controlling caries progression. These techniques which are easily applicable and do not require special equipment or training might be the basis for supporting efficacious prevention and early intervention of the disease process. Given that dental caries despite being a highly prevalent disease is now considered an ecological and non-communicable disease, these techniques may find great applicability in the public sector.

## Strengths and limitations

To the best of our knowledge, this is the first study to assess parental priorities and preferences regarding the treatment of primary teeth in Greece. In addition, it is the first study to include both traditional treatment preferences and acceptance of new minimally invasive techniques. There are very few studies in the literature that refer to parental choices in primary dentition, increasing this study’s contribution to the topic. The main limitation is that there was no clinical examination of the children to confirm parental answers and facilitate assessment of their preferences, based on the oral health status of their child. Another limitation is that participants were recruited from one public dental clinic, which despite being the only one in Athens, where 50% of the Greek population lives, is characterized by a predominance of families of lower socioeconomic backgrounds. This makes it difficult to generalize the conclusions, although participants’ characteristics are quite similar to the national average (Eurostat, 29 May 2023). Finally, given that it was a questionnaire-based study, the effect of reporting bias could not be eliminated. In future studies, a qualitative approach might give a more in depth understanding of parental preferences.

In terms of future research, more studies are needed, particularly on "traditional" treatment preferences of parents, to provide a more comprehensive picture of parental beliefs and expectations regarding dental treatment and to facilitate shared decision-making.

## Conclusions

Considering any limitations of the present questionnaire study in Greek parents/guardians regarding their children's dental treatment it has been clearly shown that:

- Age, parental education level and family status were not correlated to children’s oral hygiene habits, while sugary snacks consumption was significantly correlated with low parental education level.
- Higher parental education was also correlated with recognition of the importance of the primary dentition.
- Invasive treatment acceptance was very high for both symptomatic and asymptomatic teeth, while there was no significant preference for treatment type.
- Acceptance of SDF treatment was overall moderate and was higher among highly educated parents and parents of boys.
- Parents' knowledge and beliefs about oral health and new treatment techniques are still limited, but the opinion of the dentist is valued and accepted by the parents. These results indicate the need for dentists as individuals to educate and guide patients and parents/guardians about proper oral health practices and new treatment options but always while taking into consideration the preferences of the patient and the family.

## Data Availability

The datasets used and/or analysed during the current study are available from the corresponding author upon reasonable request.
